# Virtual Reality to Improve Breastfeeding Outcomes: A Systematic Review and Meta-Analysis

**DOI:** 10.3390/nursrep16060209

**Published:** 2026-06-22

**Authors:** Alok Raghav, Geetanjali Kalyan, Soumya Jyoti Raha, Jitendra Meena, Jogender Kumar, Praveen Kumar

**Affiliations:** 1 Department of Pediatrics, Post Graduate Institute of Medical Education and Research, Chandigarh 160012, India; alokalig@gmail.com (A.R.); soumya.j.raha@gmail.com (S.J.R.); drpkumarpgi@gmail.com (P.K.); 2 National Institute of Nursing Education (NINE), Post Graduate Institute of Medical Education and Research, Chandigarh 160012, India; geetss2@gmail.com; 3 Department of Pediatrics, All India Institute of Medical Sciences, New Delhi 110029, India; jitendra.2544aiims@gmail.com

**Keywords:** virtual reality, immersive technology, breastfeeding behavior, breastfeeding self-efficacy, breastfeeding motivation, maternal education

## Abstract

**Background**: Breastfeeding enhances infant and maternal health, but global breastfeeding rates remain suboptimal. Virtual reality (VR) emerges as a promising tool for breastfeeding education. The objective of this review was to assess the effectiveness of VR-based interventions on breastfeeding outcomes in pregnant and postpartum women. **Methods**: PubMed, Embase, Web of Science, Scopus, and CENTRAL were searched until 10 January 2026, for randomized controlled trials (RCTs) and quasi-experimental studies comparing VR-based interventions (immersive simulations, 360° videos, or head-mounted displays) with standard care or non-VR comparators in pregnant or postpartum women. Primary outcomes included breastfeeding self-efficacy, motivation, and breastfeeding technique (LATCH score). Secondary outcomes included exclusive breastfeeding rates, milk production, and maternal anxiety. Risk of bias was assessed using the RoB 2.0 and ROBINS-I tools for RCTs and non-RCTs, respectively. A random-effects meta-analysis was conducted, with results reported as mean differences (MD) or risk ratios (RR), along with 95% confidence intervals (CIs). Certainty of the evidence was assessed using the GRADE approach. **Results**: Five studies (4 RCTs and 1 quasi-experimental; *n* = 344) were included. VR improved prenatal breastfeeding self-efficacy (2 studies, MD: 13.93; 95% CI: 10.96–16.90), motivation (1 study, MD: 2.88; 95% CI: 1.66–4.10), and LATCH score (1 study, MD: 1.72; 95% CI: 1.37–2.07), and reduced time to breastfeeding initiation (1 study, MD: −22.4 min; 95% CI: −29 to −15.9), the certainty of evidence was low to very low for these outcomes. No significant effects were observed for postnatal self-efficacy, exclusive breastfeeding, or maternal anxiety. Formal assessment of publication bias could not be done. The small sample sizes for most outcomes, heterogeneity, the open-label nature of the trials, and the subjective nature of the outcomes should be considered when interpreting these results. **Conclusions**: VR-based interventions may improve process outcomes, such as prenatal breastfeeding self-efficacy, motivation, breastfeeding technique, and early breastfeeding initiation; the certainty of evidence is low to very low. Evidence for clinically important outcomes, including exclusive breastfeeding and maternal anxiety, remains inconsistent. Larger, well-designed RCTs are warranted before these interventions can be considered in routine practice.

## 1. Introduction

Breastfeeding is one of the most cost-effective and evidence-based public health interventions for reducing childhood mortality [1]. Infants and young children are more likely to survive and achieve their full developmental potential when breastfed. Beyond promoting optimal brain development, breastfeeding is essential for preventing malnutrition, infections, and early childhood mortality, and for reducing the risk of metabolic diseases later in life [2,3,4]. Mothers who breastfeed also derive substantial long-term health benefits, including reduced risks of breast and ovarian cancers, type 2 diabetes, and cardiovascular disease [4]. Furthermore, suboptimal breastfeeding results in global economic losses exceeding US$341 billion annually, approximately 0.7% of the world’s gross national income through increased healthcare expenditures, impaired child cognitive development, and lost workforce productivity [5]. Despite these well-established benefits, only about 48% of infants under six months are exclusively breastfed worldwide, far below the World Health Assembly target of 60% by 2030 [6].

Breastfeeding initiation and continuation are frequently influenced by maternal, infant, social, cultural, and healthcare-related factors. Most common barriers to breastfeeding include lack of knowledge, misinformation, low breastfeeding self-efficacy, maternal anxiety and stress, insufficient professional support, workplace constraints, and limited family support. These barriers may hamper maternal confidence for successful breastfeeding initiation and continuation. Two noteworthy clinical situations that affect breastfeeding initiation and continuation are cesarean section delivery and newborn admission to a neonatal intensive care unit (NICU). Mothers who undergo planned cesarean section are about 25% less likely to continue breastfeeding than those who deliver vaginally [7], partly due to delayed skin-to-skin contact, postoperative pain, and disruption of early mother-infant interaction. In the NICU, maternal-infant separation, the physiological immaturity of preterm infants, and the stressful environment further complicate breastfeeding. In addition to these clinical challenges, psychological factors such as maternal anxiety, stress, and reduced confidence in breastfeeding ability play a critical role [8]. These factors influence breastfeeding self-efficacy, defined as a mother’s confidence in her ability to breastfeed, which has consistently been shown to affect breastfeeding duration and exclusivity [9]. Encouragingly, many of these barriers, particularly those related to knowledge, confidence, experiential learning, and psychological support, are potentially modifiable through targeted educational and behavioral interventions [8].

Virtual reality (VR) is an emerging technology that creates immersive and interactive environments, offering new opportunities for learning and behavioral support in healthcare [10]. By allowing users to engage with realistic simulations actively, VR can enhance understanding, build confidence, and improve skill acquisition beyond conventional teaching approaches [11,12]. In the context of breastfeeding, VR may be particularly useful for addressing several modifiable barriers faced by mothers. It can provide guided demonstrations of breastfeeding techniques, allowing mothers to observe and practice positioning and latch in a supportive virtual environment [13]. At the same time, VR-based relaxation experiences may help reduce maternal stress and anxiety, which is known to influence breastfeeding confidence and milk production negatively [11,13]. Importantly, VR-based tools can also overcome practical barriers of traditional education, enabling mothers to access individualized guidance and supportive content at convenient times and across different clinical settings [12].

However, the evidence on VR-based breastfeeding interventions remains limited. We therefore conducted a systematic review and meta-analysis to evaluate the efficacy of VR-based interventions (immersive simulations, 360° videos, or head-mounted displays) on breastfeeding outcomes among pregnant and postpartum mothers.

## 2. Methods

### 2.1. Protocol Registration and Reporting

The protocol was prospectively registered with PROSPERO (CRD420261280219) (Supplementary I). The review was conducted in accordance with the Cochrane Handbook for Systematic Reviews of Interventions and reported following the Preferred Reporting Items for Systematic Reviews and Meta-Analyses (PRISMA) 2020 guidelines [14] (Supplementary II).

### 2.2. Eligibility Criteria

#### 2.2.1. Research Question, Study Design, and Participants

The research question (PICO) for this systematic review was “Does use of VR-based interventions (immersive simulations, 360° videos, or head-mounted displays) (I) compared to standard care or non-VR-based interventions (C) among pregnant and postpartum mothers (P) lead to better breastfeeding outcomes (O)?”

We included randomized controlled trials (RCTs) and quasi-experimental controlled studies that compared a VR-based intervention with a standard care or non-VR comparator. Eligible participants were pregnant women (antenatal period) or postpartum women. We include both antenatal and post-natal populations, as in real practice, it is more of a continuum of care, rather than two separate entities. Also, there is sufficient evidence that antenatal interventions have a direct impact on postnatal maternal and neonatal outcomes.

#### 2.2.2. Interventions and Comparators

The intervention was defined as any virtual reality-based intervention (head-mounted displays, 360° immersive video, or interactive simulations) with a primary focus on breastfeeding support, including education, skills training (positioning/attachment/latching), experiential rehearsal, or relaxation during milk expression. The comparator group included standard antenatal or postnatal care or non-VR-based educational or behavioral interventions.

Studies including single-arm studies, qualitative-only reports, and case series/reports were excluded. The detailed eligibility criteria are provided in Supplementary Table S1.

### 2.3. Outcomes

The outcomes were categorized into process outcomes (primary) and clinical outcomes (secondary).

These process outcomes included measures reflecting behavioral and skill-based aspects of breastfeeding, namely breastfeeding self-efficacy assessed using validated scales such as the Breastfeeding Self-Efficacy Scale (BSES) [15], Breastfeeding Self-Efficacy Scale-Short Form (BSES-SF), and prenatal self-efficacy scales [16], breastfeeding motivation (assessed using validated motivation scales) [17], and breastfeeding performance/competence assessed using scales such as LATCH (Latch, Audible swallowing, Type of nipple, Comfort, and Hold) score [18].

The secondary outcomes included the following:

*Breastfeeding-related outcomes:* exclusive breastfeeding (initiation, duration, and overall rates) and expressed breast milk volume.

*Maternal psychological and experiential outcomes*: Maternal anxiety or stress was assessed using validated instruments such as the State-Trait Anxiety Inventory (STAI), a widely used measure of state and trait anxiety [STAI] [19], and maternal satisfaction with breastfeeding was evaluated using the Maternal Breastfeeding Evaluation Scale (MBFES), which measures maternal perceptions and experiences related to breastfeeding [20].

### 2.4. Information Sources and Search Strategy

We systematically searched PubMed, Embase, Web of Science, Scopus, and CENTRAL from inception to 10 January 2026. Two authors (JK, JM) developed the database-specific search, which was peer-reviewed and refined by the other two (GK, AR) using the Peer Review of Electronic Search Strategies (PRESS) checklist [21]. We did not use any restrictions. Full, reproducible strategies are provided in Supplementary Table S2. Reference lists of relevant articles were manually screened for additional eligible studies. A forward and backward citation search was also done to identify additional studies.

### 2.5. Study Selection

All retrieved records were imported into Rayyan (Rayyan Systems, Inc., MA, USA) (https://www.rayyan.ai/), an AI-assisted systematic review platform, for deduplication and blinded screening. Following deduplication, two reviewers (JK, AR) independently screened titles and abstracts against the eligibility criteria, followed by independent full-text assessment of potentially eligible records. Disagreements at any stage were resolved through discussion and, where necessary, by consensus with a third reviewer (JM).

### 2.6. Data Extraction and Risk of Bias Assessment

Data were independently extracted by two reviewers (JK, AR) using a pre-piloted standardized spreadsheet that captured the study characteristics (design, setting, country, year), participant demographics (age, antenatal/ postnatal, neonatal characteristics, mode of delivery), interventions (type, time of administration, duration of administration, frequency), comparators (content, timing, and duration), and outcomes (primary and secondary outcomes listed above, the definitions or scales used by authors, time period of assessment). The extracted data were verified by a third reviewer (JM). Risk of bias was independently assessed by two reviewers (SJR, AR) using the Cochrane Risk of Bias tool version 2.0 (RoB 2) for RCTs [22] and ROBINS-I for non-randomized studies [23]. Disagreements were resolved by consensus with a third reviewer (JK).

### 2.7. Data Synthesis and Statistical Analysis

Meta-analyses were conducted using Review Manager 5.4 (web version) (Cochrane, United Kingdom) and were deemed feasible only if two or more similar studies reported similar outcomes. Binary outcomes were pooled as risk ratios (RRs) with 95% confidence intervals (CIs); continuous outcomes were pooled as mean differences (MDs) in case of studies that used similar, comparable outcome scales, while standardized mean differences (SMDs) with 95% CIs were reported for outcomes measured using conceptually similar but different instruments. Medians and interquartile ranges were converted to means and standard deviations using standard methods [24]. A random-effects model was specified *a priori* to account for anticipated clinical and methodological heterogeneity across included studies. Statistical heterogeneity was quantified using a combination of the I^2^ statistic, the Tau-square statistic, and Cochran’s Q test, rather than relying solely on I^2^ (particularly for continuous outcomes). Prespecified sensitivity analyses included: (1) restriction to studies at low risk of bias; and (2) reanalysis using a fixed-effect model to assess the robustness of pooled estimates. Meta-analysis was undertaken only when at least two studies reported conceptually similar outcomes using comparable outcome measures. Given anticipated clinical heterogeneity related to population characteristics, intervention type, and timing of outcome assessment, random-effects models were prespecified for all pooled analyses. The pooled estimates were complemented by narrative synthesis to facilitate interpretation of context-specific findings. To explore heterogeneity, we planned to perform subgroup analyses by intervention timing (antenatal vs. postnatal), VR intervention type, and assessment timing (1 week, 6 weeks, 6 months), provided sufficient data is available. We also planned to conduct a meta-regression by intervention duration, if the data permits. We aimed to assess publication bias using funnel plots (supplemented with Egger’s test) if enough studies (10 or more) were available for a given outcome. We followed the core GRADE approach to assess the certainty of the evidence.

## 3. Results

### 3.1. Study Selection

**Figure 1:**
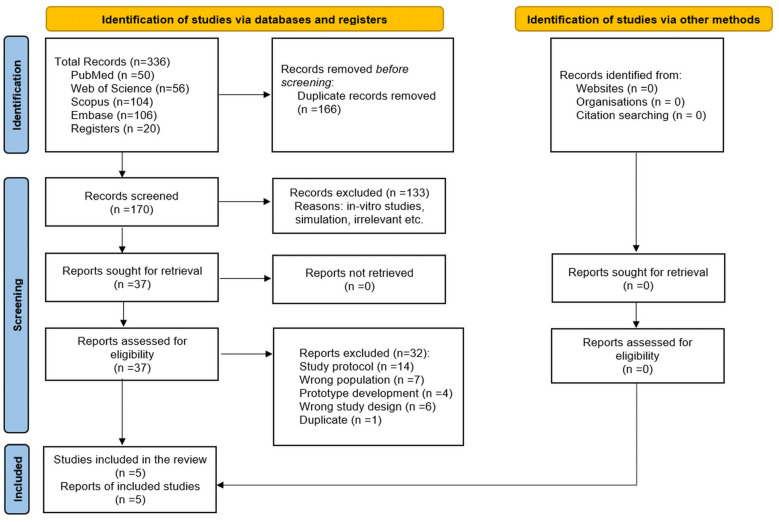
PRISMA flowchart.

We identified 335 records, of which 37 were considered eligible for full-text assessment. Of these, four RCTs and one quasi-experimental trial met the eligibility criteria (Figure 1) [13,25,26,27,28]. Most of the excluded studies were related to prototype development or were study protocols. A list of excluded studies, with reasons, is provided in Supplementary III.

### 3.2. Study Characteristics

The five studies included a total of 344 participants. All included studies were single-center (Table 1). Three studies were from Turkey, and one each from China and Colombia. Of the five studies included, three were conducted during the antenatal period [26,27,28], focusing on prenatal education and breastfeeding preparation. The remaining two studies were conducted in the postpartum period [13,25] primarily addressing breastfeeding outcomes and maternal psychological parameters after childbirth. Among the eligible studies, the three trials enrolled primiparous pregnant women in the third trimester attending prenatal care settings [26,27,28], while two included postpartum mothers (one with cesarean sections [25], another with mothers of preterm infants whose babies were admitted to the NICU [13]). The intervention included a VR-based intervention, either as a stand-alone experimental module or as an adjunct to standard counseling. At the same time, the comparator arms received the exclusive routine breastfeeding education or traditional counseling without VR technology. Most studies showed low risk of bias across key domains, with consistent concerns in outcome measurement leading to an overall judgment of “some concerns.” The quasi-experimental study demonstrated moderate to high risk of confounding and selection bias, lowering the overall certainty of the evidence (Supplementary Figure S1).

### 3.3. Primary Outcomes

#### 3.3.1. Breastfeeding Self-Efficacy

**Figure 2:**
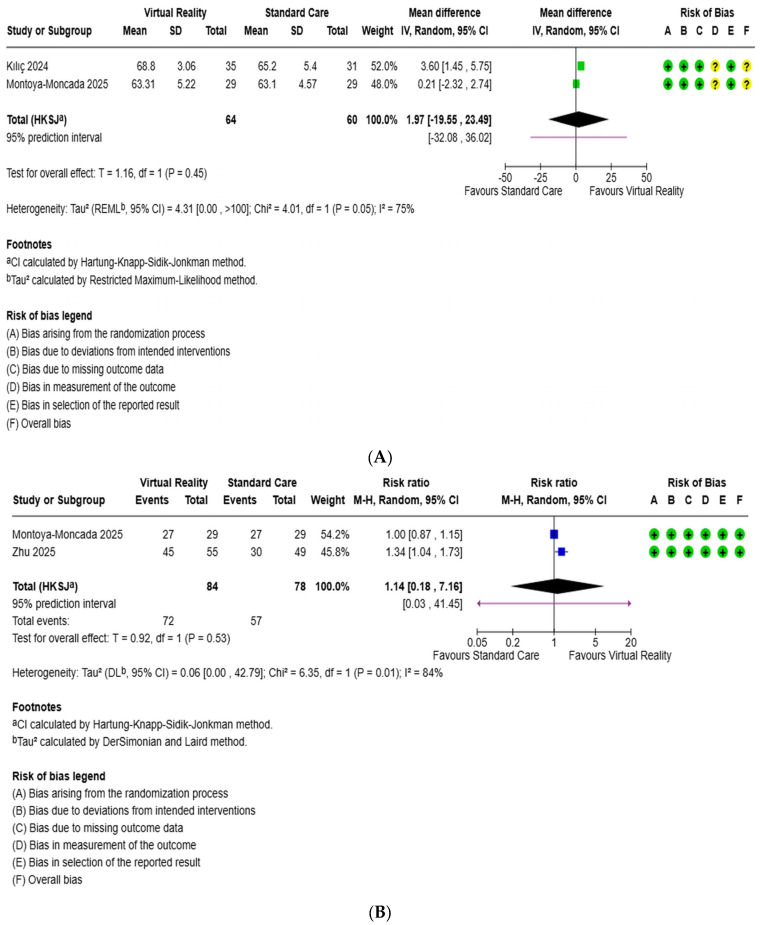

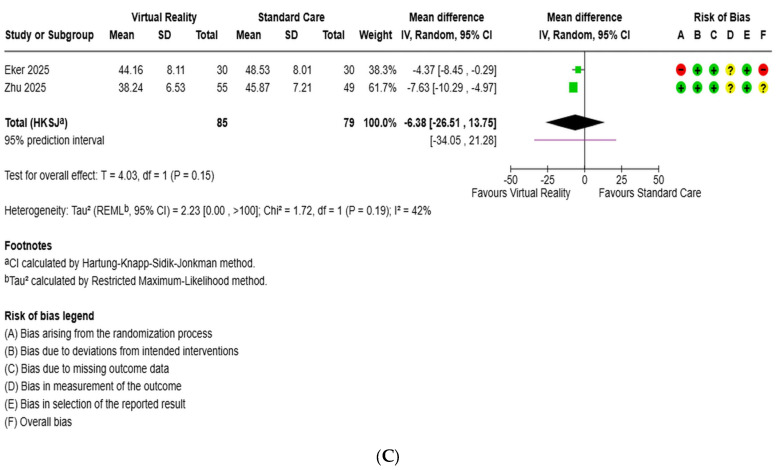
(**A**) Forest plot comparing breastfeeding self-efficacy scores between virtual reality-based interventions and control groups. (**B**) Forest plot comparing exclusive breastfeeding rates between virtual reality-based interventions and control groups. (**C**) Forest plot comparing maternal anxiety scores between virtual reality-based interventions and control groups.

Two RCTs (124 participants) reported postnatal self-efficacy and observed no statistically significant difference in self-efficacy scores on the Breastfeeding Self-Efficacy Scale (MD: 1.97; 95% CI: −19.55 to 23.49, Tau-square-4.3) [25,26] (Figure 2A). The study that administered the intervention in the post-partum period found that breastfeeding self-efficacy was significantly higher in the VR-based intervention group; however, its clinical significance is very limited (merely a 3-point difference). In clinical practice, a 5- to 10-point increase on the BSES is considered clinically meaningful. In contrast, there was no significant difference when the intervention was administered in the antenatal period. These findings suggest that, in principle, the clinically meaningful effect was not observed in either period (low-certainty evidence).

Whereas one RCT (52 participants) reported prenatal self-efficacy (prenatal BSES, not pooled) and observed a statistically significant improvement with the VR-based intervention (MD: 13.93; 95% CI: 10.96 to 16.90) [28]. This difference is also clinically meaningful (a 7–12-point difference is considered clinically significant), but the certainty of evidence was low.

#### 3.3.2. Motivational Attitudes

One RCT (52 participants) assessed breastfeeding motivation using the Breastfeeding Motivation Scale (BMS) and found slightly better motivation levels with VR-based intervention (MD: 2.88; 95% CI: 1.66 to 4.10, low-certainty) [28].

#### 3.3.3. Observed Breastfeeding Technique Proficiency

In an RCT (66 participants) examining the breastfeeding technique using the LATCH score, the VR-based intervention group showed slightly higher LATCH scores compared with controls (MD: 1.72; 95% CI: 1.37 to 2.07, low-certainty) [25].

### 3.4. Secondary Outcomes

#### 3.4.1. Breastfeeding Initiation (Time to First Breastfeed)

One RCT (104 participants) provided data on time to first breastfeeding [27]. Infants in VR-based education received their first breastfeed 22.4 min (95% CI: 15.9 to 26) earlier than in standard care (low-certainty evidence).

#### 3.4.2. Exclusive Breastfeeding

Two RCTs (162 participants) reported exclusive breastfeeding rates at different postpartum time points. In both studies, the intervention was administered during the antenatal period. Montoya-Moncada et al. reported no significant difference in exclusive breastfeeding rates at one week postpartum, whereas Zhu et al. observed higher exclusive breastfeeding rates in the VR group at six weeks postpartum (Figure 2B) [26,27]. The study assessing exclusive breastfeeding rates at one week showed no statistically significant difference between the two groups (RR: 1.0; 95%CI: 0.87 to 1.15) [26]. However, at 6 weeks postpartum, exclusive breastfeeding rates were significantly higher in the VR group (RR: 1.34; 95% CI: 1.04 to 1.73) [27]. The pooled analysis demonstrated no statistical difference between the VR-based intervention and standard care (RR 1.14; 95% CI: 0.18–7.16; I2 = 84%, low-certainty evidence). The substantial heterogeneity across studies is likely due to differences in the timing of outcome assessment, and the small sample sizes limit confidence in the pooled estimate. Therefore, current evidence regarding the effect of VR on exclusive breastfeeding remains inconclusive.

#### 3.4.3. Effect on Milk Expression

One quasi-experimental study (30 participants) assessed the effect of VR-based intervention in the postnatal period on breast milk production in [13]. In participants receiving VR-based education, post-intervention milk volume was significantly higher on days 4–6 (57.00 ± 39.80 mL/day) than on days 1–3 (38.61 ± 38.33 mL/day) during standard care sessions (*p* < 0.05). However, it must be noted that, physiologically, the milk volume on days 4–6 will be higher than on days 1–3. Therefore, causal attribution of the observed increase to the VR intervention is not possible.

Another study where an intervention was done in the antenatal period compared the 48 h milk secretion volume between two groups [27]. In the intervention groups (VR), the 48 h milk secretion volume was significantly higher (118.42 ± 35.67 vs. 96.83 ± 32.94 mL, *p* = 0.002) than that in the control group [27].

Due to differences in intervention timing and assessment time points, the results were not pooled.

#### 3.4.4. Maternal Anxiety

Two studies (134 participants) assessed maternal anxiety using the State-Trait Anxiety Inventory (STAI) and observed no significant difference in the two groups (MD: −6.38; 95% CI: −26.51 to 13.75) [13,27] (Figure 2C). While individual studies differed in direction and magnitude of effect, pooled analysis demonstrated no statistically significant reduction in maternal anxiety. Therefore, available evidence does not currently support a consistent anxiolytic effect of VR interventions in breastfeeding populations.

### 3.5. Additional Analysis

We planned to do a subgroup analysis by intervention timing (antenatal vs. postnatal), VR intervention type, and assessment timing (1 week, 6 weeks, 6 months). Due to the limited number of studies, the formal subgroup analysis was not considered feasible or meaningful. Most trials reported only short-term outcomes [13,25,26,27,28]. We also planned to conduct a meta-regression by intervention duration, but the data did not permit meaningful meta-regression. As and when possible, we stratified the analysis as per intervention timing (antenatal or postnatal).

Sensitivity analysis using fixed-effect modeling yielded effects with similar direction and magnitude across the major outcomes, indicating the robustness of the primary findings. As all except one study were of some concern, precluding meaningful sensitivity analysis on that aspect, too.

### 3.6. Certainty of Evidence and Publication Bias

The certainty of the evidence was low to very low across most outcomes, primarily due to risk of bias, imprecision, and small sample size. A detailed description of downgrading is provided in the Summary of Findings table (Table 2). Downgrading was mainly due to a small sample size, wide CIs, and some clinical heterogeneity. A smaller number of studies per outcome precluded a formal assessment of publication bias (*n* = 5 overall; ≤2 per pooled analysis). Consequently, the possibility of publication bias cannot be excluded. This limitation should be taken into account when interpreting the findings.

## 4. Discussion

This systematic review and meta-analysis synthesized evidence evaluating VR-based interventions for breastfeeding support. There is limited evidence from five small studies. Although some studies reported improvements in process-related outcomes such as antenatal breastfeeding self-efficacy, motivation, breastfeeding technique, and time to first breastfeeding, the evidence was derived from small studies with substantial methodological and clinical heterogeneity. Furthermore, clinically important outcomes, including exclusive breastfeeding rates and maternal anxiety, showed inconsistent findings across studies. Therefore, the current evidence should be considered exploratory and hypothesis-generating rather than confirmatory.

Virtual reality-based interventions mainly target proximal outcomes (knowledge, confidence, self-efficacy, motivation, procedural competence), which may support earlier initiation and better technique. Distal outcomes, such as exclusive breastfeeding duration, maternal anxiety, and sustained behavior, are shaped by complex biological, psychosocial, and health-system factors beyond brief educational interventions. Therefore, gains in proximal measures should not be taken as evidence of long-term clinical benefit. The available evidence suggests that VR interventions may have a greater influence on process and educational outcomes, including confidence, motivation, and breastfeeding technique, than on longer-term clinical outcomes. While these findings are encouraging, improvements in surrogate measures do not necessarily translate into sustained breastfeeding behavior or improved maternal and infant health outcomes. Therefore, the clinical significance of the observed effects remains uncertain.

The improvement in time to first breastfeeding is clinically relevant. Earlier initiation is a well-recognized determinant of breastfeeding success, and the observed reduction (≈22 min) suggests that antenatal VR exposure may improve preparedness and reduce hesitation in the immediate postpartum period. Alzaheb et al. linked early initiation of breastfeeding to the delivery-related and contextual factors (maternal employment, rooming-in, pre-lacteal feeding practices, etc.) [29]. Immersive rehearsal during the antenatal period may reduce postpartum uncertainty and function as a training and counseling tool, facilitating earlier initiation. Similarly, improvements in LATCH scores reflect better procedural competence, supporting the role of VR as a skills-training adjunct rather than a standalone behavioral intervention.

In contrast, the absence of consistent improvement in postnatal self-efficacy and exclusive breastfeeding should be interpreted within the broader causal architecture of breastfeeding behavior. Sustained breastfeeding outcomes are influenced by multiple interacting factors, including maternal stress, physical recovery, infant health, healthcare practices, family support, and socio-cultural context [30]. A brief, time-limited VR intervention, particularly when delivered antenatally, cannot reasonably be expected to modify these structural and contextual determinants. Thus, the lack of effect on longer-term outcomes likely reflects limitations in the intervention’s scope rather than the modality’s ineffectiveness.

The observed pattern in self-efficacy outcomes further supports this interpretation. While antenatal self-efficacy improved significantly in one study, pooled estimates were imprecise and inconsistent. This suggests that VR may enhance anticipatory or task-specific confidence. Still, this effect may attenuate once mothers encounter real-world challenges such as pain, fatigue, infant behavior, and environmental constraints. In this context, VR appears to influence preparedness, but not necessarily adaptation over time.

Educational psychology models propose that VR learning operates through complementary cognitive (information processing, attentional focus, working memory) and affective (emotional engagement, intrinsic motivation, and experiential presence) pathways [31]. Immersive VR further augments learning through what is called the “Presence Theory”: a heightened sense of realism, emotional investment, and investment in the activity. By providing structured, repetitive, life-like exposures in a psychologically relaxed setting, VR may reduce cognitive load before real-life breastfeeding, facilitating skill encoding and anticipatory confidence, consistent with Bandura’s self-efficacy framework [32].

From a physiological perspective, stress and anxiety can inhibit oxytocin-mediated milk ejection through sympathetic activation [5]. The reduction in maternal anxiety observed with VR-based interventions offers a plausible pathway for improved early lactation responses, particularly in high-stress settings such as post-cesarean recovery or NICU admission. However, sustained milk production depends on complex hormonal regulation, infant demand, and continued support, and real-world data on the impact of VR on actual breast milk volume remain limited.

Systematic reviews of VR in healthcare education (including surgical training, nursing, and Cardiopulmonary resuscitation training) consistently show improvements in short-term procedural skills, knowledge, and learner confidence [33,34,35]. However, these studies also highlight small sample sizes, heterogeneity, short-term assessment, and limited evidence of downstream patient-level benefits, a pattern that mirrors our findings.

Reviews on virtual lactation support (telehealth-based counseling) report modest increases in exclusive breastfeeding at 1 and 6 months, but these interventions differ substantially from immersive VR [36,37]. To our knowledge, no prior systematic review has specifically assessed the available evidence on VR-based interventions for breastfeeding; our synthesis fills this gap. Single studies reported improvements in breastfeeding motivation, LATCH scores, and milk production, and therefore require independent replication before confidence in these effects can be established. Furthermore, pooled analyses of clinically important outcomes such as exclusive breastfeeding and maternal anxiety did not demonstrate a consistent benefit. Consequently, the current evidence suggests potential effects on selected educational and process-related outcomes rather than definitive improvements in long-term breastfeeding outcomes.

### Limitations

Several limitations constrain the conclusions that can be drawn from this synthesis. The evidence base comprises only five single-center studies enrolling 344 participants from predominantly middle-income settings, which affects generalizability. The limited number of studies also precluded robust subgroup analyses and failed to provide definitive conclusions regarding the effectiveness of different VR modalities or implementation strategies. Differences in the intervention period (antenatal and postnatal), intervention objectives, VR platforms, exposure duration, and timing of outcome assessment may have contributed to the substantial heterogeneity. Consequently, pooled effect estimates should be viewed as exploratory summaries rather than precise estimates of intervention effectiveness. Participant blinding is inherently impossible in immersive VR trials, introducing performance and detection bias that plausibly inflate effect estimates for subjective outcomes, including self-efficacy and anxiety. These aspects were considered during the GRADE process, which led to reduced confidence in the findings, as reflected in low to very low certainty of the evidence. None of the included studies assessed breastfeeding outcomes beyond 42 days postpartum, making it impossible to conclude the outcomes that matter most (exclusive breastfeeding at 3 and 6 months), and rendering the current evidence base insufficient to inform guideline recommendations.

Though we could not formally assess the small-study effect, publication bias remains possible: emerging technologies like VR are often evaluated in small, single-center studies, and positive findings are more likely to be published, which can inflate early effect estimates.

The policy implication of this review is therefore prospective rather than prescriptive. Since the studies are conducted in limited settings with no cost analysis, they may not be directly applicable in low- and middle-income countries (LMICs). Implementing VR-based interventions in these settings would require careful consideration of resource requirements, including VR equipment, infrastructure, training needs, and direct and indirect costs. Such considerations are especially important because the burden of preterm birth is greatest in LMICs, where the need for effective antenatal interventions is most pronounced.

At present, the available evidence does not support integrating VR into routine antenatal care. However, it does provide a rationale for investing in adequately powered, multicenter randomized controlled trials that include LMIC settings. Future studies should employ standardized VR protocols, assess longer-term maternal and neonatal outcomes, incorporate economic evaluations, and report results stratified by parity, clinical setting, and mode of delivery to better inform policy and practice.

## 5. Conclusions

The current evidence for VR-based breastfeeding interventions remains limited and preliminary. Available studies suggest that VR may improve selected process-related outcomes, including antenatal breastfeeding self-efficacy, motivation, breastfeeding technique, and time to breastfeeding initiation. However, the current evidence does not yet support clinical or policy recommendations. Adequately powered, multicenter trials with standardized protocols, follow-up extending to six months postpartum, and evaluation of cost-effectiveness are needed before immersive VR can be considered for integration into routine maternity care.

## Figures and Tables

**Table 1:** Baseline Characteristics and outcome assessment (*n* = 5 studies).

Author (Year); Country	Study Design/Setting	Study Population	Sample Size (Intervention/Control)	Baseline Characteristics	Intervention	Comparator	Outcomes	Assessment Time Point
***Postpartum period***								
Kılıç et al. (2024) [25]; Turkey NCT06256822	*Design:* RCT *Setting:* Postpartum ward	Postpartum women after cesarean section	35/35	*Maternal:* Age 26–33 years. *Obstetric:* Cesarean section (100%); term pregnancies. *Neonatal:* Term neonates; sex distribution comparable.	**Type:** VR-based breastfeeding education **Device/Platform:** Shinecon 3D VR goggles **Content:** 3-D instructional breastfeeding video **Dose/Duration/Timing:** Single 10 min session, early postpartum	Standard breastfeeding education	Breastfeeding efficacy (BSES-SF) Breastfeeding success (LATCH score) Breastfeeding problems	4 and 24 h
Eker et al. (2025) [13]; Turkey NCT06153628	*Design:* Quasi-experimental (Pre and Post intervention design) *Setting:* NICU	Postpartum women whose babies are preterm and admitted to NICU (30–37 weeks)	30/30	*Maternal:* Mean age 27.9 ± 5.6 years. *Obstetric:* Preterm delivery (30–37 weeks). *Neonatal:* Mean birth weight 1777 ± 658 g.	**Type:** VR-based relaxation intervention **Device/Platform:** Oculus Quest 2 **Content:** Immersive nature and underwater relaxation environments **Dose/Duration/Timing:** 10 min daily for 3 consecutive postpartum days (days 4–6)	Pre-intervention baseline (self-controlled)	Maternal anxiety (State Trait Anxiety Inventory scale) Expressed breast-milk volume (mL)	Baseline (days 1–3), postpartum (days 4–6)
***Antenatal period***								
Montoya-Moncada et al. (2025) [26]; Colombia NCT06800521	*Design:* RCT *Setting:* Hospital	Pregnant women in the third trimester (Antenatal)	29/29	**Maternal:** 48.3% college education; ~50% prior breastfeeding experience. **Obstetric:** Third trimester; 58.6% had no living children.	**Type:** Mixed-reality breastfeeding education plus traditional counseling **Device/Platform:** Mixed-reality headset with doll simulator **Content:** Immersive breastfeeding education combined with hands-on simulation **Dose/Duration/Timing:** One structured antenatal session lasting for 60 min	Traditional breastfeeding counseling	Breastfeeding Score obtained on BSES-SF and the MBFESscales Exclusive breastfeeding rates at 1 week	Baseline, 1-week postpartum
Zhu et al. (2025) [27]; China	*Design*: RCT *Setting*: Community health center	Elderly primiparous pregnant women in the third trimester (Antenatal)	55/49	**Maternal:** Mean age ~37 years; **Obstetric:** Singleton term pregnancy; primiparous.	Individualized childbirth education program, including VR-based education **Type:** VR-assisted prenatal education **Device/Platform:** HTC Vive Pro 2 **Content:** Locally developed and validated immersive childbirth and breastfeeding preparation module. **Dose/Duration/Timing:** Single session (40 min) at approximately 32 weeks’ gestation	Routine prenatal classes (4 classes at 20, 28, 32, and 36 weeks of gestation, each lasting 60 min.	Maternal anxiety and self-efficacy Exclusive BF rate Time to first BF 48 h milk volume	Baseline, post-delivery, post-discharge (42 days postpartum)
Ertaş et al. (2026) [28]; Turkey NCT06697769	*Design*: RCT *Setting*: Family health center	Primiparous Pregnant women (Antenatal)	26/26	**Maternal:** Mean age 28.7 ± 4.1 vs. 26.8 ± 3.6 years; education and employment comparable. **Obstetric:** Gestational age ≈ 36 weeks; primiparous.	**Type:** VR breastfeeding experience/simulation **Device/Platform:** Oculus Quest 2 **Content:** Interactive breastfeeding scenarios (hospital and home settings) **Dose/Duration/Timing:** Two sessions of 5 min each, antenatal period	Routine antenatal education	Prenatal breastfeeding self-efficacy (Prenatal Breastfeeding Self-Efficacy Scale) Postpartum breastfeeding motivation (Breastfeeding Motivation Scale)	Baseline (antenatal), post-intervention, early postpartum

Abbreviations: BF—Breastfeeding; BSES-SF—Breastfeeding Self-Efficacy Scale-Short Form; g—Gram; MBFES—Maternal Breastfeeding Evaluation Scale; mL—Milliliter; NCT—National Clinical Trial Identifier; NICU—Neonatal Intensive Care Unit; RCT—Randomized Controlled Trial; VR—Virtual Reality.

**Table 2:** Summary of Findings.

Outcome	No. of Studies (Participants)	RR/MD (95% CI)	Certainty of Evidence (GRADE)	Explanation
Postnatal Breastfeeding Self-efficacy	2 RCTs (124 participants)	1.97 (−19.55 to 23.49)	⊕⊕◯◯ Low	Downgraded for serious imprecision (wide 95% CI, small sample size) and some risk of bias (1 level each)
Antenatal breastfeeding self-efficacy	1 RCT (52 participants)	13.93 (10.96 to 16.90)	⊕⊕◯◯ Low	Downgraded for not meeting OIS criteria (small sample) and some risk of bias (1 level each)
Breastfeeding motivation	1 RCT (52 participants)	2.88 (1.6 to 4.10)	⊕⊕◯◯ Low	Downgraded for very serious imprecision (by 2 levels) and single-study evidence
Breastfeeding success (LATCH scores)	1 RCT (66 participants)	1.72 (1.37 to 2.07)	⊕⊕◯◯ Low	Downgraded for not meeting OIS (by 2 levels)
Exclusive breastfeeding	2 RCTs (162 participants)	1.14 (0.18 to 7.16)	⊕⊕◯◯ Low	Downgraded for very serious imprecision and not meeting OIS (by 2 levels)
Time to first breastfeeding	1 RCT (104 participants)	−22.42 (−28.95 to −15.89)	⊕⊕◯◯ Low	Downgraded for very serious imprecision (by 2 levels)
Maternal anxiety	2 studies (164 participants) Quasi-experimental (01) RCT (01)	−6.38 (−26.51 to 13.75)	⊕◯◯◯ Very low	Downgraded for serious risk of bias, imprecision, and indirectness (1 level each)

Abbreviations: CI—Confidence Interval; GRADE—Grading of Recommendations Assessment, Development and Evaluation; LATCH—Latch, Audible swallowing, Type of nipple, Comfort, Hold; MD—Mean Difference; OIS: Optimal Information Size, RCT—Randomized Controlled Trial; RR—Risk Ratio.

## Data Availability

All data used in this systematic review and meta-analysis are publicly available. No new datasets were generated or analyzed for this study.

## Supplementary Materials

The following supporting information can be downloaded at: https://www.mdpi.com/article/10.3390/nursrep16060209/s1.

## Public Involvement Statement

Patients and members of the public were not involved in the design, conduct, reporting, or dissemination of this systematic review and meta-analysis.

## Guidelines and Standards Statement

This systematic review and meta-analysis was conducted and reported in accordance with the Preferred Reporting Items for Systematic Reviews and Meta-Analyses (PRISMA 2020) statement. Where applicable, the review protocol was developed following the recommendations of the Cochrane Handbook for Systematic Reviews of Interventions.
