# Supplementary material for: Virtual Reality to Improve Breastfeeding Outcomes: A Systematic Review and Meta-Analysis
Source: Nurs Rep. 2026 Jun 22;16(6):209. doi: 10.3390/nursrep16060209 (PMC13304627; doi:10.3390/nursrep16060209)
Supplement: Supplementary file 1 [file nursrep-16-00209-s001.zip › 1. Supplementary I_PROSPERO_VR.pdf]

NIHR | National Institute for Health and Care Research

PROSPERO
International prospective register of systematic reviews

Effect of Virtual Reality on Breastfeeding: A Systematic Review

Jogender Kumar, Jitendra Meena, Alok Raghav

Citation
Jogender Kumar, Jitendra Meena, Alok Raghav. Effect of Virtual Reality on Breastfeeding: A Systematic Review . PROSPERO 2026 CRD420261280219. Available from https://www.crd.york.ac.uk/PROSPERO/view/CRD420261280219.

REVIEW TITLE AND BASIC DETAILS

Review title
Effect of Virtual Reality on Breastfeeding: A Systematic Review

Condition or domain being studied
Breast Milk Finding; Virtual Reality

Rationale for the review
Breastfeeding is a key determinant of neonatal and maternal health, yet many women experience barriers related to inadequate preparation, anxiety, pain, surgical delivery, or neonatal illness requiring NICU admission. Educational and psychosocial interventions are central to improving breastfeeding outcomes, but traditional modalities may have limited engagement and retention.

Virtual reality (VR)–based interventions provide immersive, interactive learning environments that may enhance skill acquisition, motivation, confidence, and emotional regulation. Recent primary studies underscore growing interest in this approach. Despite this emerging body of evidence, no up-to-date systematic review has specifically synthesized the effects of VR or metaverse-based interventions on breastfeeding outcomes. Existing breastfeeding education reviews do not address immersive digital technologies. A comprehensive systematic review is therefore needed to inform clinical practice, maternal education strategies, and future research.

Review objectives
To systematically identify, critically appraise, and synthesize evidence on the effectiveness of virtual reality (VR)–based interventions on breastfeeding outcomes, including self-efficacy, motivation, breastfeeding performance, milk production, exclusivity, and maternal psychological outcomes.

Keywords

Breastfeeding; Virtual reality; Postpartum

Country

India

ELIGIBILITY CRITERIA

Population

Included

Participants: Pregnant women, postpartum women, and mothers of preterm or sick infants (including NICU mothers).

Intervention(s) or exposure(s)

Included

Virtual Reality

Any virtual reality–based intervention, including:

1. Immersive VR using head-mounted displays
2. Non-immersive or semi-immersive VR
3. Metaverse-based breastfeeding education
4. VR simulations or experiential breastfeeding modules

Interventions may be delivered antenatally or postnatally.

Comparator(s) or control(s)

Included

PICO tags selected: Usual Care; Education; Counseling; Family Support; Breastfeeding Education; Breastfeeding Support

- Standard care
- Conventional breastfeeding education (verbal, written, video)
- Face-to-face counseling or peer support
- Any non-VR intervention

Excluded

Any use of VR in any form

Study design

Both randomized and nonrandomized study types will be included.

Included

RCTs preferred. If studies are not available/insufficient; we will also include non-randomized controlled studies.

Excluded

Exclusions: Qualitative studies, case reports and series, Studies with no control group, editorials, narrative reviews.

Context

Breastfeeding is a key determinant of neonatal and maternal health, yet many women experience barriers related to inadequate preparation, anxiety, pain, surgical delivery, or neonatal illness requiring NICU admission. Educational and psychosocial interventions are central to improving breastfeeding outcomes, but traditional modalities may have limited engagement and retention.

Virtual reality (VR)–based interventions provide immersive, interactive learning environments that may enhance skill acquisition, motivation, confidence, and emotional regulation. Recent primary studies underscore growing interest in this approach:

Kilic et al. (2024) demonstrated improved breastfeeding self-efficacy and LATCH scores among post-cesarean mothers receiving VR-based training.

Eker et al. (2025) reported increased expressed breast milk volume and reduced maternal anxiety among NICU mothers exposed to VR-supported lactation education.

Ertaş et al. (2026) showed significantly higher breastfeeding self-efficacy and breastfeeding motivation among pregnant women exposed to a VR breastfeeding experience compared with standard education.

NCT07078825 and other registered trials are evaluating metaverse-based or immersive VR breastfeeding education programs.

Despite this emerging body of evidence, no up-to-date systematic review has specifically synthesized the effects of VR or metaverse-based interventions on breastfeeding outcomes. Existing breastfeeding education reviews do not address immersive digital technologies. A comprehensive systematic review is therefore needed to inform clinical practice, maternal education strategies, and future research.

TIMELINE OF THE REVIEW

Date of first submission to PROSPERO

07 January 2026

Review timeline

Start date: 7 January 2026. End date: 7 June 2026.

Date of registration in PROSPERO

07 January 2026

AVAILABILITY OF FULL PROTOCOL

Availability of full protocol

A full protocol has been written and uploaded to PROSPERO. The protocol will be made available after the review is completed.

SEARCHING AND SCREENING

Search for unpublished studies
Only published studies will be sought.

Main bibliographic databases that will be searched
The main databases to be searched are *CENTRAL - Cochrane Central Register of Controlled Trials*, *CINAHL - Cumulative Index to Nursing and Allied Health Literature*, *Embase.com*, *MEDLINE*, *PubMed* and *Scopus*.

Search language restrictions
There are no language restrictions.

Search date restrictions
There are no search date restrictions.

Other methods of identifying studies
Other studies will be identified by: *looking through all the articles that cite the papers included in the review ("snowballing" or forward citation searching)*, *reference list checking (backward citation searching)* and *searching trial or study registers*.

Link to search strategy
A full search strategy is available in the full protocol as described in the *Availability of full protocol* section

Selection process
Studies will be screened independently by at least two people (or person/machine combination) with a process to resolve differences.

Other relevant information about searching and screening
None

DATA COLLECTION PROCESS

Data extraction from published articles and reports
Data will be extracted independently by at least two people (or person/machine combination) with a process to resolve differences.

Authors will be asked to provide any required data not available in published reports.

Study risk of bias or quality assessment
Risk of bias will be assessed using: *Cochrane RoB-2* and *ROBINS-I*

Data will be assessed independently by at least two people (or person/machine combination) with a process to resolve differences.

Additional information will be sought from study investigators if required information is unclear or unavailable in the study publications/reports.

Reporting bias assessment
Risk of bias due to missing results will not be assessed

Certainty assessment

Certainty of findings will not be assessed

OUTCOMES TO BE ANALYSED

Main outcomes

Primary:

• Breastfeeding self-efficacy (e.g., BSES, BSES-SF, prenatal self-efficacy scales)

• Breastfeeding motivation (validated motivation scales)

• Breastfeeding performance/competence (e.g., LATCH score)

Additional outcomes

Secondary:

• Expressed breast milk volume/amount

• Exclusive breastfeeding rates, initiation, and duration

• Maternal anxiety/stress (e.g., STAI)

• Maternal satisfaction with breastfeeding experience (e.g., MBFES)

• Breastfeeding knowledge and intervention satisfaction/acceptability

PLANNED DATA SYNTHESIS

Strategy for data synthesis

If 2 or more studies provide similar data then we will pool the data. We will do random-effects meta-analysis where appropriate; narrative synthesis otherwise. Heterogeneity assessed using I^2, τ^2 and a combination of other measures. We will use RevMan for data analysis. .

CURRENT REVIEW STAGE

Stage of the review at this submission

Review stage	Started	Completed
Pilot work		
Formal searching/study identification		
Screening search results against inclusion criteria		
Data extraction or receipt of IPD		
Risk of bias/quality assessment		
Data synthesis		

Review status

The review is currently planned or ongoing.

Publication of review results

Results of the review will be published in English.

REVIEW AFFILIATION, FUNDING AND PEER REVIEW

Review team members

Dr Jogender Kumar (review guarantor and contact) Post Graduate Institute of Medical Education and Research Chandigarh. India.

No conflict of interest declared.

Dr Jitendra Meena. AIIMS New Delhi. India.

No conflict of interest declared.

Dr Alok Raghav. PGIMER CHANDIGARH. India.

No conflict of interest declared.

Named contact

Dr Jogender Kumar (jogendrayadv@gmail.com). Post Graduate Institute of Medical Education and Research Chandigarh. India.

Review affiliation

PGIMER Chandigarh; India

Funding source

Review has no funding and no agreed support from an academic institution and is done in authors' own time.

Peer review

The protocol has been peer reviewed and approved by team members

ADDITIONAL INFORMATION

Review conflict of interest

Declared individual interests are recorded under team member details.. No additional interests are recorded for this review.

Medical Subject Headings

Humans; Milk; Motivation; Self Efficacy; Virtual Reality

SIMILAR REVIEWS

Check for similar records already in PROSPERO

PROSPERO identified a number of existing PROSPERO records that were similar to this one (last check made on 7 January 2026). These are shown below along with the reasons given by that the review team for the reviews being different and/or proceeding.

- Effectiveness of virtual reality technologies compared to conventional technologies in cardiopulmonary resuscitation training in lay populations: a systematic review protocol. [published 11 November 2025] [CRD420251184110]. The review was judged **not to be similar**
- Integrating Virtual Reality and Artificial Intelligence in Health Care: A Systematic Review [published 3 September 2025] [CRD420251127099]. The review was judged **not to be**

similar

- Effects of Virtual Reality–Based Interventions on Caregiving Competence, Empathy, and Self-Efficacy among Dementia Caregivers: A Systematic Review and Meta-Analysis of Randomized Controlled Trials [published 28 July 2025] [CRD420251114461]. The review was judged **not to be similar**
- Effects of Virtual Reality on Pain, Anxiety, and Fear During Port-a-Cath Needle Procedures in Cancer Patients: A Systematic Review and Meta-Analysis of Randomized Controlled Trials [published 12 December 2025] [CRD420251251849]. The review was judged **not to be similar**
- The effect of perioperative virtual reality interventions on pain, anxiety, and related outcomes in adults undergoing hand or wrist surgery: a systematic review and meta-analysis [published 2 January 2026] [CRD420251269092]. The review was judged **not to be similar**

PROSPERO version history

- Version 1.0, published 07 Jan 2026

Disclaimer

The content of this record displays the information provided by the review team. PROSPERO does not peer review registration records or endorse their content.

PROSPERO accepts and posts the information provided in good faith; responsibility for record content rests with the review team. The guarantor for this record has affirmed that the information provided is truthful and that they understand that deliberate provision of inaccurate information may be construed as scientific misconduct.

PROSPERO does not accept any liability for the content provided in this record or for its use. Readers use the information provided in this record at their own risk.

Any enquiries about the record should be referred to the named review contact
