# Supplementary material for: Virtual Reality to Improve Breastfeeding Outcomes: A Systematic Review and Meta-Analysis
Source: Nurs Rep. 2026 Jun 22;16(6):209. doi: 10.3390/nursrep16060209 (PMC13304627; doi:10.3390/nursrep16060209)
Supplement: Supplementary file 1 [file nursrep-16-00209-s001.zip › 3. Supplementary Table S1.pdf]

Supplementary Table S1: Inclusion and Exclusion Criteria

Framework	Inclusion criteria	Exclusion criteria
**Population**	1. Pregnant women<br>2. Postpartum women	1. Participants other than pregnant or postpartum women
**Intervention**	1. Virtual Reality (VR) based breastfeeding education or support interventions<br>2. Immersive VR using head-mounted displays<br>3. Non-immersive or semi-immersive VR interventions<br>4. Metaverse-based breastfeeding education programs<br>5. VR simulations or experiential breastfeeding training modules delivered antenatally or postnatally	1. Interventions not using VR technology<br>2. Traditional education methods without VR component (verbal, written, video only)
**Comparator**	1. Standard care<br>2. Conventional breastfeeding education (verbal, written, video-based)<br>3. Face-to-face counseling or peer support<br>4. Any non-VR educational or supportive intervention	1. Studies without a comparator group<br>2. Comparisons involving VR vs VR only
**Outcome**	**Primary outcomes**<br>• Breastfeeding self-efficacy<br>• Breastfeeding motivation<br>• Breastfeeding attitude<br>• Breastfeeding success (LATCH score)<br>• Breastfeeding problems<br>**Secondary outcomes**<br>• Exclusive breastfeeding<br>• Time to first breastfeeding<br>• Expressed breast milk volume<br>• Maternal anxiety	1. Studies not reporting breastfeeding-related outcomes
**Study Design**	1. Randomized Controlled Trials (RCTs)<br>2. Non-randomized controlled studies (Must have comparator arm)	1. Qualitative studies<br>2. Case reports or case series<br>3. Studies without control group
