# Supplementary material for: Virtual Reality to Improve Breastfeeding Outcomes: A Systematic Review and Meta-Analysis
Source: Nurs Rep. 2026 Jun 22;16(6):209. doi: 10.3390/nursrep16060209 (PMC13304627; doi:10.3390/nursrep16060209)
Supplement: Supplementary file 1 [file nursrep-16-00209-s001.zip › 4. Supplementary Table S2_Search strategy.pdf]

Supplementary Table S2: Search Strategy for Electronic Databases and Registers_VR in breastfeeding

Database	Query	Search Terms	Number of Hits
PubMed	#1 Population	("Breast Feeding"[Mesh] OR "Lactation"[Mesh] OR "Milk, Human"[Mesh] OR breastfeeding[tiab] OR "breast feeding"[tiab] OR lactation[tiab] OR "breast milk"[tiab] OR colostrum[tiab] OR "human milk"[tiab] OR "Lactation Disorders"[Mesh] OR "infant feeding"[tiab] OR ((pregnan*[tiab] OR antenatal[tiab] OR prenatal[tiab] OR postpartum[tiab] OR postnatal[tiab]) AND (breastfeeding[tiab] OR lactation[tiab] OR "breast milk"[tiab])))	165101
	#2 Intervention	("Virtual Reality"[Mesh] OR "virtual reality"[tiab] OR VR[tiab] OR metaverse[tiab] OR "head-mounted display"[tiab] OR "head mounted display"[tiab] OR HMD[tiab] OR ((immersive[tiab] OR simulation[tiab] OR experiential[tiab]) AND (virtual[tiab] OR reality[tiab])))	50213
	#3	#1 AND #2	**50**
Embase	#1 Population	'breast feeding'/exp OR 'breast feeding' OR 'lactation'/exp OR 'lactation' OR 'human milk'/exp OR 'human milk' OR 'lactation disorder'/exp OR 'lactation disorder' OR breastfeeding:ti,ab OR 'breast feeding':ti,ab OR lactation:ti,ab OR 'breast milk':ti,ab OR colostrum:ti,ab OR 'human milk':ti,ab OR 'infant feeding':ti,ab OR ((pregnan*:ti,ab OR antenatal:ti,ab OR prenatal:ti,ab OR postpartum:ti,ab OR postnatal:ti,ab) AND (breastfeeding:ti,ab OR lactation:ti,ab OR 'breast milk':ti,ab))	212429
	#2 Intervention	'virtual reality'/exp OR 'virtual reality':ti,ab OR vr:ti,ab OR metaverse:ti,ab OR 'head mounted display'/exp OR 'head-mounted display':ti,ab OR 'head mounted display':ti,ab OR hmd:ti,ab OR ((immersive:ti,ab OR simulation:ti,ab OR experiential:ti,ab) AND (virtual:ti,ab OR reality:ti,ab))	75196
	#3	#1 AND #2	**106**
Web of Science	#1 Population	(breastfeeding OR "breast feeding" OR lactation OR "breast milk" OR colostrum OR "human milk" OR "infant feeding" OR ((pregnan* OR antenatal OR prenatal OR postpartum OR postnatal) AND (breastfeeding OR lactation OR "breast milk"))) (Topic)	148642
	#2 Intervention	("virtual reality" OR VR OR metaverse OR "head-mounted display" OR "head mounted display" OR HMD OR ((immersive OR simulation OR experiential) AND (virtual OR reality))) (Topic)	115621
	#3	#1 AND #2	**56**
Scopus	#1 Population	TITLE-ABS-KEY (breastfeeding OR "breast feeding" OR lactation OR "breast milk" OR colostrum OR "human milk" OR "infant feeding" OR ((pregnan* OR antenatal OR prenatal OR postpartum OR postnatal) AND (breastfeeding OR lactation OR "breast milk")))	235726
	#2 Intervention	TITLE-ABS-KEY ("virtual reality" OR VR OR metaverse OR "head-mounted display" OR "head mounted display" OR HMD OR ((immersive OR simulation OR experiential) AND (virtual OR reality)))	401477
	#3	#1 AND #2	**104**
ClinicalTrials.gov	#1 Population	breastfeeding OR lactation OR Breast Milk Expression OR Human Milk/Breastfeeding	3227
	#2 Intervention	virtual reality OR VR OR immersive OR metaverse OR head-mounted display	4353
	#3	#1 AND #2	**19**
CENTRAL	ID Search Hits		

	#1 MeSH descriptor: [Breast Feeding] explode all trees 3033<br>#2 MeSH descriptor: [Lactates] explode all trees 4084<br>#3 MeSH descriptor: [Milk, Human] explode all trees 1534<br>#4 MeSH descriptor: [Breast Milk Expression] explode all trees 57<br>#5 MeSH descriptor: [Virtual Reality] explode all trees 1675<br>#6 MeSH descriptor: [Smart Glasses] explode all trees 26<br>#7 MeSH descriptor: [Augmented Reality] explode all trees 145<br>#8 MeSH descriptor: [Virtual Reality Exposure Therapy] explode all trees 434<br>#9 #1 OR #2 OR #3 OR #4 8069<br>#10 #5 OR #6 OR #7 OR #8 2108<br>#11 #9 AND #10 **1**
