# Supplementary material for: Virtual Reality to Improve Breastfeeding Outcomes: A Systematic Review and Meta-Analysis
Source: Nurs Rep. 2026 Jun 22;16(6):209. doi: 10.3390/nursrep16060209 (PMC13304627; doi:10.3390/nursrep16060209)
Supplement: Supplementary file 1 [file nursrep-16-00209-s001.zip › 5. Supplementary III_excluded studies.pdf]

List of excluded studies with reasons

S. No	Title	Year	Reasons for exclusion
1	The Effect of Breastfeeding Experience Presented to Pregnant Women With Virtual Reality Glasses on Breastfeeding Self-efficacy and Breastfeeding Motivation: A Randomized Controlled Trial	2024	Duplicate
2	EffÄ±cÄ±ency Of HybrÄ±d Based SÄ±mulatÄ±on Method In BreastfeedÄ±ng EducatÄ±on Of MÄ±dwÄ±fery Students	2024	Simulation based Protocol
3	VR FOR STRESS REDUCTION AND SLEEP HEALTH IMPROVEMENT AMONG PERINATAL WOMEN: INSIGHTS FROM THE NURTURING MOMS STUDY	2024	Different outcome,Different study design
4	I am Lifted Above the World: utilizing VR for stress reduction among perinatal women of color	2024	Different study design
5	Evaluating the effect of a virtual reality digital therapeutic on maternal stress among expectant and postpartum Black and Latina mothers: a protocol paper	2024	Study Protocol
6	Learning via avatars: An experiential journey for medical imaging students	2012	Different population
7	Evaluation of the effect of breastfeeding counseling education model developed in metaverse on breastfeeding counseling skills, knowledge and empathy level	2025	Nursing student,Different population
8	Diving into Immersive Education: How Midwifery Students Experience 360-Degree Videos for Breastfeeding Support	2025	Nursing student,Different population
9	VR Breastfeeding: A Preliminary User Experience Study comparing Head and Eye Tracking Interactions	2025	Prototype development
10	Playful Reflection: Impact of Gamification on a Virtual Reality Simulation of Breastfeeding	2023	Prototype development
11	Virtual simulation on breastfeeding and nipple-areolar lesions: prototype development and validation	2023	background article,Prototype development
12	Virtual Lactation Education in a Pandemic	2022	participants were interns
13	Virtual Feed: Design and Evaluation of a Virtual Reality Simulation Addressing the Lived Experience of Breastfeeding	2022	Prototype development
14	Challenges and Opportunities for Playful Technology in Health Prevention: Using Virtual Reality to Supplement Breastfeeding Education	2022	Different population,Different study design
15	Virtual Experiential Learning: A Description of an Internship Framework That Engages Students to Build Public Health Competencies	2021	Different population |
16	Virtual Feed: A Simulated Breastfeeding Experience in Virtual Reality	2021	Prototype development
17	The Morphology of Dignity: Service Storytelling and Prototypes for a Service Design Tool	2019	Different population,Different study design
18	Assessing a virtual baby feeding training system	2010	population,Prototype development
19	The playability evaluation of virtual baby feeding application	2010	Prototype development
20	Feasibility, Acceptability and Effects of Virtual Reality Distraction for Management of Stress in Mothers Expressing Their Maternal Milk (VR-MOM)	2023	Protocol
21	The Effect of Metaverse- and Virtual Reality-Supported Education in the Digital Breastfeeding Museum on Mothers' Breastfeeding Knowledge Level, Self-efficacy, and Success	2025	Protocol

22	The Effect of the "VR Baby-Pump" Application Applied to Mothers Who Gave Birth Preterm on Their Breast Milk Expression Experience, Transition Process, and Milk Quantity: Randomized Controlled Study	2023	proticol
23	The Effect of Virtual Reality Application on Anxiety and Milk Quantity in Mothers Who Milk Their Babies by Milking	2025	Protocol
24	The Effect of Virtual Reality Glasses Applied to Mothers Whose Babies Are in the Neonatal Intensive Care Unit on the Level of Anxiety and the Amount of Breast Milk	2023	Protocol
25	The Effect of Viewing Images of Fetus to Pregnant Women With Virtual Reality Glasses on Birth Pain, Perception and Anxiety During Labor	2020	Different outcome,Mainly for labour pain
26	Effect of Technology-Based Breastfeeding Training After Cesarean on Breastfeeding Success and Self-Efficacy of Mothers	2024	Protocol
27	Evaluation of the Effect of the Breastfeeding Counseling Education Model Developed in the Metaverse Universe on the Counseling Skills, Knowledge and Empathy Levels of Nursing Students	2024	Protocol
28	Effects of VE, Focus, Expressive Touch on Pain, Anxiety, Breastfeeding and Mobilization in Women Delivering by Cesarean Section	2024	Protocol
29	The Effect of Antenatal Breastfeeding Education Prepared With Virtual Reality Technology on Breastfeeding Self-Efficacy and Breastfeeding Success of Primiparous Mothers	2023	Protocol
30	The Effect of Breast Massage and Virtual Reality Application on Lactation in Postpartum Women: A Randomized Controlled Trial	2025	Protocol
31	Effects of Integrative Sensory-Supported Virtual Reality and Gamified Digital Breastfeeding Education on Mothers' Breastfeeding Success, Self-Efficacy and Adaptation	2024	Protocol
32	Mixed Reality as a Health Literacy Strategy for Breastfeeding Education: A Randomized Controlled Trial	2025	Protocol
