# Supplementary material for: Virtual Reality to Improve Breastfeeding Outcomes: A Systematic Review and Meta-Analysis
Source: Nurs Rep. 2026 Jun 22;16(6):209. doi: 10.3390/nursrep16060209 (PMC13304627; doi:10.3390/nursrep16060209)
Supplement: Supplementary file 1 [file nursrep-16-00209-s001.zip › 6. Supplementary Figure S1.pdf]

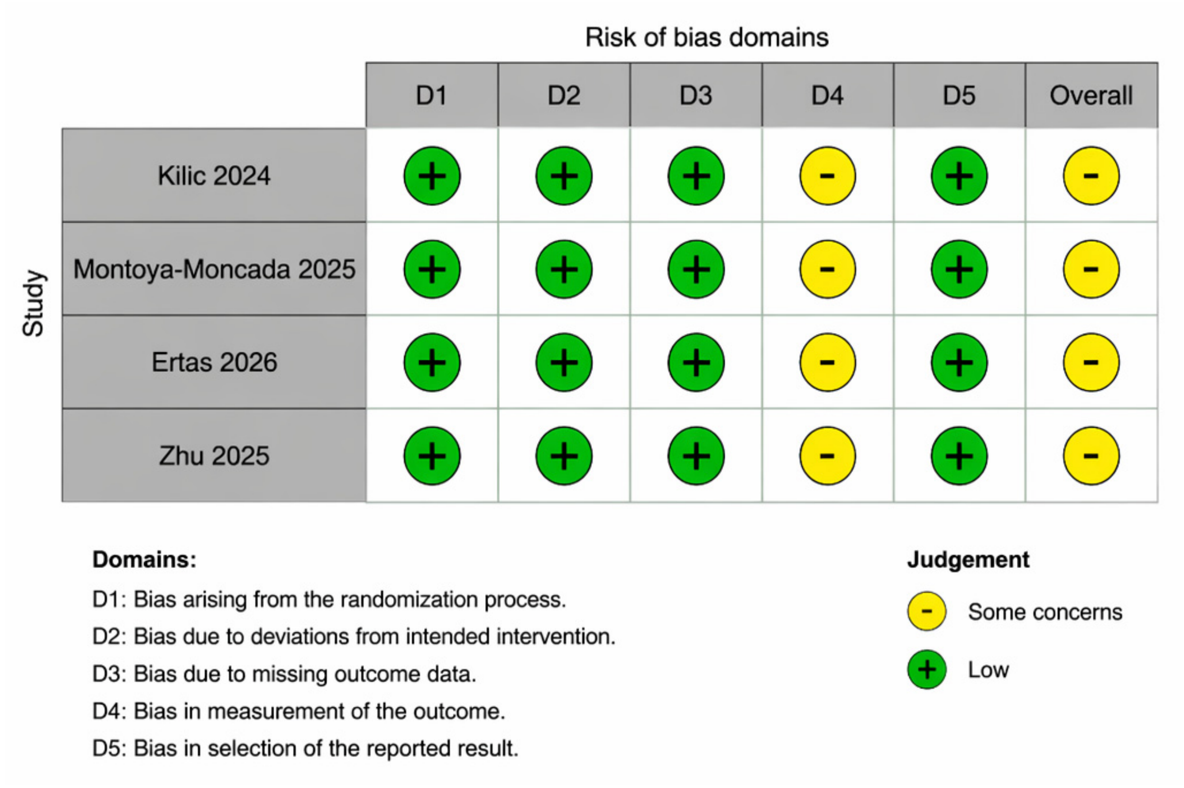

(**A**)

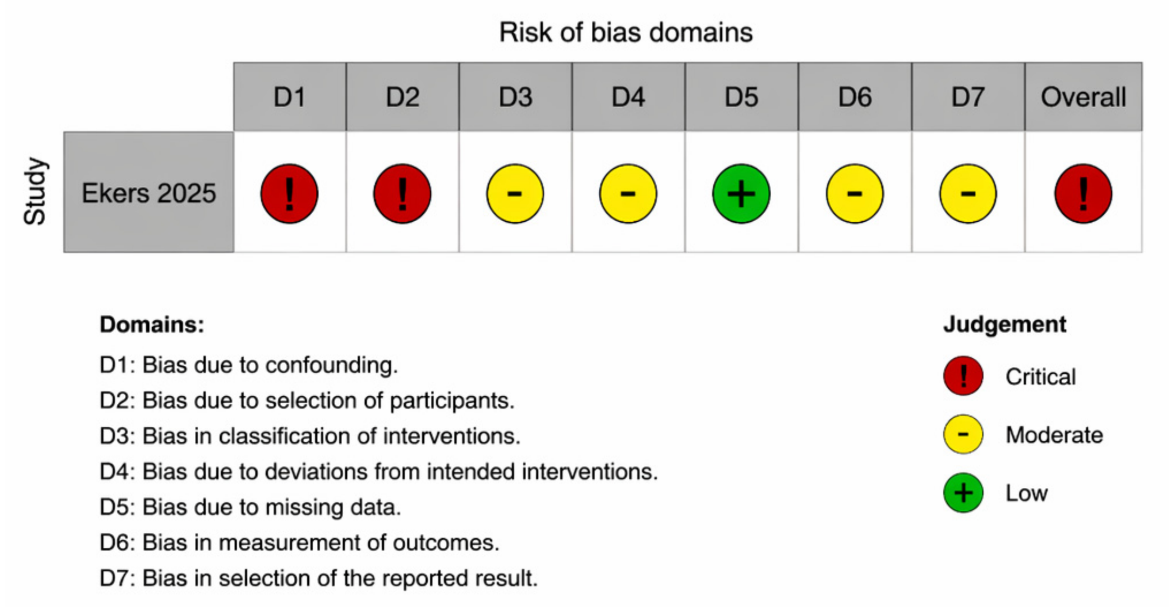

(**B**)

Supplementary Figure S1. (**A**) Risk of Bias assessment using Traffic Plot using ROB 2.0; (**B**) Risk of Bias assessment using Traffic Plot using ROBINS-I
